# Adaptive Path Integral Diffusion: AdaPID

**DOI:** 10.3390/e28050492

**Published:** 2026-04-24

**Authors:** Michael Chertkov, Hamidreza Behjoo

**Affiliations:** Program in Applied Mathematics, Department of Mathematics, University of Arizona, Tucson, AZ 85721, USA; hamidreza.behjoo@gmail.com

**Keywords:** generative models, diffusion models, diffusion bridge, optimal transport, path integrals, stochastic optimal control, schedule optimization, Gaussian mixtures, sampling diagnostics

## Abstract

Harmonic Path Integral Diffusion (H-PID) provides an analytically tractable framework for sampling from a target density $p^{\left(\mathrm{tar}\right)}\left(x\right)\propto \exp\left(-E\left(x\right)\right)$. H-PID can be viewed as a diffusion bridge model solving a stochastic optimal transport problem from a δ-density at t=0 to the target density at t=1. The dynamics are governed by a controlled stochastic differential equation, and the corresponding variational stochastic optimal transport objective combines a time-dependent quadratic potential, $\beta_{t}{∥x_{t}∥}^{2}/2$, with a kinetic control cost, $∥u\left(t;x_{t}\right){∥}^{2}/2$. The focus of this paper is the design of the temporal stiffness protocol $\beta_{t}$, which enables explicit control of intermediate sampling dynamics when the terminal density is fixed. We exploit the central advantage of H-PID—its integrability—which yields an explicit representation of the optimal control in terms of the target density and Green functions of the associated linear forward and backward diffusion-in-a-potential problems. Our main contribution is to convert this integrable structure into a practical methodology for protocol optimization. Specializing to piecewise-constant stiffness schedules and Gaussian-mixture targets, we develop two complementary optimization principles: The first is a deterministic one, relying on explicit evaluation of the dynamic marginals, and exemplified on a velocity-gradient-sensitivity objective, which provides a computationally controlled framework for optimizing transport regularity and stiffness. The second is a stochastic one, implemented via sampling, and exemplified on sharpness-based temporal-memory objective regularized to favor transitions within a prescribed time window that targets the temporal organization of the sampling path. These two objectives illuminate different aspects of the same protocol-design problem. The velocity-gradient-sensitivity objective serves as a clean methodological backbone and supports interpretable optimization and scaling studies. The sharpness-based objective reveals that schedule quality is target-dependent, and that the dependence on β is not universal: different target geometries may favor different stiffness regimes and qualitatively different transient organizations. Examples with low- and moderate-dimensional Gaussian mixtures demonstrate that the proposed approach can control not only the terminal sampling accuracy but also the transient evolution of probability mass, while remaining computationally light and theoretically transparent.

## 1. Introduction

Diffusion-based sampling methods [1,2,3,4,5] have recently emerged as one of the most powerful paradigms for generative modeling and stochastic inference. A number of closely related frameworks have been developed in this context, including denoising diffusion probabilistic models (DDPMs) [2], score-based diffusion models [3], Schrödinger bridge formulations [4], and flow-based generative models [5]. Despite their different formulations, these approaches share a common conceptual objective: to construct a stochastic or deterministic transport process that progressively transforms a simple reference distribution into a complex target distribution.

A fundamental feature shared by diffusion-based generative methods is that the target distribution alone does not uniquely determine the dynamics of the sampling process. There typically exists a large family of stochastic dynamics whose terminal distribution coincides with the desired target distribution. Consequently, the design of the diffusion dynamics itself becomes a nontrivial modeling choice.

Most existing work evaluates such constructions primarily in terms of their ability to reproduce the target distribution asymptotically. However, this criterion alone does not distinguish between the many possible transport dynamics that achieve the same terminal distribution. An important question therefore arises: **how should one choose among these admissible dynamics?**

In this work, we argue that the choice of dynamics should be guided not only by the correctness of the terminal distribution but also by the properties of the **transport trajectory** connecting the reference and target distributions. Different dynamics correspond to different transport paths and can lead to substantially different transient sampling behavior. This motivates the development of systematic criteria for evaluating diffusion dynamics beyond asymptotic correctness.

The Path Integral Diffusion (PID) framework introduced in [6] provides a particularly convenient setting for studying such questions. PID can be viewed as a diffusion bridge formulation in which the sampling process is constructed as a controlled stochastic trajectory that transports point distribution to a target distribution in unit time. Within this formulation, the score function appears naturally as an optimal control that steers the diffusion between the two distributions.

A distinctive feature of PID, generalizing the path integral control [7,8] to diffusive sampling, is that the resulting control problem is analytically integrable. Specifically, the optimal control (equivalently, the score function) admits an explicit representation in terms of a convolution between the target distribution and a ratio of Green functions associated with a pair of forward and backward linear partial differential equations. In the Harmonic PID construction, these Green functions can be written in closed form, making the transport dynamics analytically tractable while still allowing for nontrivial target distributions.

This integrable diffusion bridge structure provides a powerful analytic framework for studying diffusion dynamics. In particular, it allows one to explore how different choices of the transport protocol influence the behavior of the sampling trajectory, while maintaining an explicit connection to the target distribution.

In the original work on Harmonic Path Integral Diffusion (H-PID) [6] we introduced a constant stiffness protocol and demonstrated that the resulting dynamics admits a useful analytic structure that enables detailed analysis of the sampling process. While constant schedules provide a convenient baseline, they are not necessarily optimal for finite-time sampling, and they are also not sufficiently flexible for exploring details of transient dynamics, which can depend strongly on how the stiffness parameter varies over time.

Motivated by these observations, we ask the following question: once the terminal target law is fixed, how much freedom remains in the dynamics of sample generation, and can this freedom be used in a systematic and interpretable way? Our answer is affirmative in the analytically tractable setting of Harmonic Path Integral Diffusion (PID), where the diffusion dynamics are driven by a time-dependent quadratic potential and the target distribution is a Gaussian Mixture Model (GMM). In this setting, one can explicitly parameterize a stiffness protocol, evaluate multiple notions of schedule quality, and optimize the protocol so as to shape intermediate-time behavior rather than merely the endpoint.

We formulate a practical workflow for protocol design in Harmonic PID: how to parameterize schedules, how to distinguish deterministic and stochastic objectives, how to optimize them, and how to interpret the resulting nontrivial schedules. The central message is not simply that different objectives lead to different schedules, although this is true and important. Rather, the main point is that Harmonic PID provides a rich and controllable methodology for shaping sample-generation dynamics in a way that is both theoretically transparent and computationally efficient.

The GMM/Harmonic PID setting is particularly attractive because it provides exact access to the time-dependent marginals $p_{t}\left(x\right)$. This creates a clean separation between two classes of protocol diagnostics. One-time quantities, such as spread or sensitivity proxies based on the control field, can be evaluated deterministically from exact marginals. In contrast, two-time observables that explicitly couple the current state to the endpoint remain genuinely pathwise and must be evaluated stochastically from trajectories. This separation is conceptually important and practically useful: it allows us to combine deterministic exact-marginal calculations with controlled stochastic estimation of temporal-memory observables.

Within this framework we study piecewise-constant (PWC) stiffness schedules and develop two complementary optimization principles: The first is deterministic and is based on a velocity-gradient-sensitivity (VGS) criterion—defined later in Equation (11)—that measures control regularity and dynamical stiffness. The second is stochastic and is based on a dynamic sharpness of the transient objective regularized by the requirement for the transition to occur at a prescribed time in the middle of the temporal interval—defined later in Equation (13). Together, they provide two concrete routes to schedule design. They also reveal that controlling the transient dynamics is not only possible but sufficiently rich to generate nontrivial, interpretable protocols, including higher-dimensional PWC schedules.

The examples in this paper should therefore be read as illustrations of a broader methodology. We do emphasize concrete low-dimensional and moderately high-dimensional benchmarks, because these are necessary to make the discussion explicit, reproducible, and delivering a sharp experimental message and a clear statement of scaling capabilities. However, the intended contribution is the methodology itself: an analytically controlled, computationally light, and conceptually flexible framework for protocol optimization in diffusion-bridge-based sampling.

## 2. Setting the Stage: Problem Formulation

We study finite-time diffusion-based transport from a point source to a prescribed target law. The basic optimization problem is

(1) $$\begin{array}{cc} u_{t}^{*}\left(x;\beta_{0\to 1}\right) & =\;\arg\min_{u_{0\to 1}}\int_{0}^{1}E\;\left[\frac{1}{2}{∥u_{t}∥}^{2}+V_{t}\left(x\right)\right]dt, \end{array}$$

(2) $$\begin{array}{cc} \mathrm{subject}\;\mathrm{to}\;dx_{t} & =u_{t}\left(x_{t}\right)\;dt+dW_{t},\;t\in \left[0,1\right],\\ x_{0} & =0,\;x_{1}∼p^{\left(\mathrm{tar}\right)}, \end{array}$$

where $x_{t}\in R^{d}$ and the target density

(3) $$p^{\left(\mathrm{tar}\right)}\left(y\right)={\left(Z^{\left(\mathrm{tar}\right)}\right)}^{-1}\exp\left(-E\left(y\right)\right),\;Z^{\left(\mathrm{tar}\right)}≐\int \exp\left(-E\left(y^{\prime }\right)\right)\;dy^{\prime },$$

is defined up to a normalization factor (partition function).

The drift (or control) field $u_{t}\left(x\right)$ in Equation (2) plays the role of a time-dependent drift (in the terminology of fluid mechanics) or score (in the terminology of generative AI) that transports probability mass from the initial point source at t=0 to the target law at t=1. The central question of this paper is not whether such a transport can be constructed—this is guaranteed by the PID framework—but how the time-dependent protocol can be designed so as to shape the **transient** dynamics of sampling.

### 2.1. Harmonic PID with Time-Dependent Stiffness

Throughout this paper, we work with a quadratic confining potential

(4) $$V_{t}\left(x\right)=\frac{\beta_{t}}{2}{∥x∥}^{2},\;\beta_{0\to 1}≐{\left\{\beta_{t}\right\}}_{t\in [0,1]}.$$

The scalar stiffness protocol $\beta_{t}$ is the main design variable in this paper. In [6], β was taken to be constant. Here, we allow it to vary in time, thereby introducing a controlled tradeoff between exploration and contraction along the sampling path.

In the experiments, we restrict attention to piecewise-constant (PWC) schedules:

(5) $$\beta_{t}=\beta^{\left(k\right)},\;t\in \left[t_{k-1},t_{k}\right),\;k=1,\ldots ,K_{\beta }.$$

This family is expressive enough to produce nontrivial schedules, while remaining low-dimensional enough to be optimized robustly with modest computational cost. It also preserves the analytic tractability of Harmonic PID.

The key structural fact is that, for any choice of protocol $\beta_{0\to 1}$, the associated stochastic optimal transport problem admits a closed-form solution for the optimal control:

(6) $$\begin{array}{cc} u_{t}^{*}\left(x;\beta_{0\to 1}\right)\; & =\nabla_{x}\log\;\left(Z^{\left(\mathrm{probe}\right)}\left(t;x;\beta_{0\to 1}\right)\right), \end{array}$$

(7) $$\begin{array}{cc} Z^{\left(\mathrm{probe}\right)}\left(t;x;\beta_{0\to 1}\right)\; & ≐\int_{R^{d}}q^{\left(\mathrm{probe}\right)}\left(y|t;x;\beta_{0\to 1}\right)\;dy, \end{array}$$

(8) $$\begin{array}{cc} q^{\left(\mathrm{probe}\right)}\left(y|t;x;\beta_{0\to 1}\right)\; & ≐p^{\left(\mathrm{tar}\right)}\left(y\right)\;\exp\;\left(-\Delta (t;x;y;\beta_{0\to 1})\right), \end{array}$$

(9) $$\begin{array}{cc} \Delta (t;x;y;\beta_{0\to 1})\; & ≐-\log\frac{G_{t}^{\left(-\right)}\left(x|y;\beta_{0\to 1}\right)}{G_{1}^{\left(+\right)}\left(y|0;\beta_{0\to 1}\right)}. \end{array}$$

Here, $q^{\left(\mathrm{probe}\right)}\left(y|t;x;\beta_{0\to 1}\right)$ may be interpreted as an unnormalized density of terminal state *y* conditioned on observing state *x* at the earlier time *t*, and $G_{t}^{\left(\pm \right)}$ denotes the forward/backward Green functions of the auxiliary linear PDEs associated with the harmonic potential. The main significance of Equations (6)–(9) is that they make the dependence of the controlled dynamics on the protocol $\beta_{0\to 1}$ explicit. Thus, the protocol is not a passive parameter but a genuine design variable that can be optimized to shape the intermediate dynamics of sample generation. Further technical details, including the specialization to PWC schedules, can be found in Appendix A.

### 2.2. Why Gaussian Mixtures?

Gaussian-mixture (GM) targets play a central role in this work, not because we wish to restrict attention to toy examples, but because they provide an exceptional degree of analytic and computational control. In the H-PID setting, Gaussian-mixture targets allow explicit representations for the following:

- The forward and backward Green-function messages;
- The optimal control field;
- The time-dependent marginal density $p_{t}\left(x\right)$;
- Several moment-based quantities derived from these marginals.

As a result, the full marginal law $p_{t}\left(x\right)$ is available explicitly at every time *t*. This is a major computational advantage, because it allows deterministic evaluation (in terms of simple integrals or elementary functions) of all **one-time** observables, $E∥f\left(x_{t}\right){∥}^{2}$ or sensitivity measures derived from the control field.

At the same time, not all quantities of interest collapse to one-time exact-marginal expressions. Temporal-memory observables such as $\hat{A}\left(t\right)$ explicitly couple the current state to the endpoint and, therefore, remain genuinely pathwise, multi-time quantities. This distinction between one-time exact-marginal observables and two-time path observables is one of the main methodological themes of this paper. It is what ultimately motivates our use of two complementary optimization principles: one deterministic and one stochastic. Additional technical details on the Gaussian-mixture simplifications are given in Appendix C.

### 2.3. What Is Being Optimized?

The object being optimized in this paper is the stiffness protocol $\beta_{0\to 1}$, parameterized through the finite-dimensional PWC coefficients $\beta^{\left(k\right)}$. We refer to this as “outer” optimization in order to distinguish it from the inner stochastic optimal transport problem that defines the optimal control for a fixed protocol. The point is not to re-optimize the terminal target law, which remains fixed throughout, but rather to optimize **how** the target is assembled over time.

Accordingly, the outputs of interest are not limited to a single best scalar parameter. They include the following:

- Constant schedules, used as simple and interpretable baselines;
- Nontrivial time-dependent PWC schedules;
- Inn higher-dimensional studies, families of optimized schedules that vary systematically with dimension or target complexity.

The emphasis throughout is on finite-budget intermediate-time behavior. We are interested in how target structure emerges in time, how sensitive the resulting controlled dynamics are, and how the protocol β(t) can be designed so as to shape this transient evolution in an interpretable way. This viewpoint leads naturally to the two optimization principles introduced next: a deterministic exact-marginal objective based on control sensitivity, and a stochastic pathwise objective based on temporal memory.

## 3. Methodology for Schedule Design

This section describes how stiffness schedules are parameterized, evaluated, and optimized once the H-PID problem is fixed. The central methodological distinction is between objectives that can be evaluated deterministically from exact marginals and objectives that remain intrinsically pathwise and, therefore, require stochastic estimation. This distinction leads naturally to two complementary optimization principles: a deterministic objective based on velocity-gradient sensitivity, and a stochastic objective based on temporal sharpness.

### 3.1. Protocol Parameterization

Throughout this paper, we work with piecewise-constant (PWC) stiffness schedules:

(10) $$\beta_{t}=\beta^{\left(k\right)},\;t\in \left[t_{k-1},t_{k}\right),\;k=1,\ldots ,K_{\beta }.$$

The number of intervals $K_{\beta }$ is kept small. In the experiments below, we typically use four intervals, which is sufficient to produce nontrivial schedules while keeping the optimization low-dimensional and interpretable. This low-dimensional parameterization is important both computationally and conceptually: it allows the resulting protocols to be optimized robustly and still remain understandable as explicit temporal design choices.

### 3.2. One-Time Versus Two-Time Observables

A central methodological distinction in this paper is between one-time and two-time observables.

#### 3.2.1. One-Time Observables

These depend only on the current marginal law $p_{t}\left(x\right)$ and can therefore be evaluated deterministically once exact marginals are available. The main examples in this work are as follows:

- Control regularity and sensitivity measures derived from the spatial Jacobian of the control field;
- Spread-like moment quantities such as $E[∥x_{t}{∥}^{2}]$;
- Exact-marginal energy- or entropy-type quantities.

#### 3.2.2. Two-Time Observables

These couple states at different times along the trajectory and, therefore, do not reduce to a one-time marginal quantity. The main example in this paper is the temporal-memory function $\hat{A}\left(t\right)$, which couples the current state to the terminal state and must therefore be estimated from simulated trajectories.

This distinction governs the entire schedule-design methodology. Whenever a quantity is intrinsically one-time, we evaluate it deterministically from exact marginals. Only when a quantity is genuinely pathwise do we resort to stochastic trajectory-based estimation.

### 3.3. Deterministic Optimization Principle: Velocity-Gradient Sensitivity

Our first optimization principle is deterministic and is based on the sensitivity of the controlled dynamics to perturbations in state space. Following the viewpoint developed in [9] and related stochastic-interpolant work [10,11], the spatial variation in the velocity field provides a useful proxy for stability, stiffness, and numerical tractability. Schedules that reduce excessive local amplification are therefore desirable.

This motivates the **velocity-gradient-sensitivity** objective

(11) $$J_{\mathrm{VGS}}\left(\beta \right)=\int_{0}^{1}E\left[∥\nabla_{x}u_{t}^{\left(*\right)}{\left(x;\beta \right)∥}_{F}^{2}\right]\;dt,$$

where ${∥\cdot ∥}_{F}$ denotes the Frobenius norm. The role of $J_{\mathrm{VGS}}$ is to penalize excessively stiff or irregular control fields and thereby favor smoother, more stable transport dynamics.

The important practical point is that $J_{\mathrm{VGS}}$ is built from one-time exact-marginal quantities. In the Gaussian-mixture setting, these quantities can be evaluated explicitly at each time. As a result, optimization of the VGS objective is deterministic and does not require Monte Carlo sampling.

### 3.4. Stochastic Optimization Principle: Regularized Transition Sharpness

Our second optimization principle is stochastic and is based on the temporal-memory observable introduced in [6]:

(12) $$\begin{array}{c} \hat{A}\left(t;\beta_{0\to 1}\right)≐\frac{E_{p^{\left(\mathrm{path}\right)}}\;\left[\hat{y}\;{\left(t;x\left(t\right);\beta_{0\to 1}\right)}^{⊤}x_{1}\right]}{E_{p^{\left(\mathrm{path}\right)}}\;\left[∥x_{1}{∥}^{2}\right]}=\frac{\sum_{m=1}^{M}\hat{y}\;{\left(t;x^{\left(m\right)};\beta_{0\to 1}\right)}^{⊤}x_{1}^{\left(m\right)}}{\sum_{m=1}^{M}{∥x_{1}^{\left(m\right)}∥}^{2}}. \end{array}$$

This quantity measures how strongly the current state reflects the eventual terminal organization, and it may be interpreted as a pathwise temporal-memory observable. As observed in [6], $\hat{A}\left(t;\beta_{0\to 1}\right)$ typically increases with time and, in some regimes, can exhibit a comparatively sharp transition from unstructured to target-informed behavior.

These properties motivate the sharpness objective

(13) $$\hat{S}\left(\beta \right)=\int_{0}^{1}\left(1-\left|2\hat{A}\left(t;\beta \right)-1\right|\right)\;dt.$$

This objective favors trajectories in which the target structure builds up in a temporally organized way.

Unlike $J_{\mathrm{VGS}}$, the sharpness objective is intrinsically multi-time. It cannot be reduced to exact-marginal deterministic evaluation and must therefore be estimated stochastically from trajectory ensembles.

On its own, $\hat{S}\left(\beta \right)$ may favor extreme stiffness regimes. To bias the transition toward a prescribed temporal window, we introduce a regularized version:

(14) $${\hat{S}}^{\left(\mathrm{reg}\right)}\left(\beta \right)=\hat{S}\left(\beta \right)+\lambda^{\left(\mathrm{trans}\right)}{\left|t^{*}\left(\beta \right)-t^{\left(\mathrm{trans}\right)}\right|}^{2},$$

where $t^{*}\left(\beta \right)$ denotes the empirically defined transition time associated with $\hat{A}\left(t;\beta \right)$, and $t^{\left(\mathrm{trans}\right)}$ is the desired transition time. In the current implementation, the regularization is two-sided: both too-early and too-late transitions are penalized relative to the chosen target time.

### 3.5. Experimental Methodology

All experiments in this paper follow the same implementation philosophy: keep the protocol space low-dimensional and interpretable, evaluate what can be computed exactly at the density level, estimate genuinely pathwise quantities only when necessary, and organize optimization through a stable hierarchical refinement.

#### 3.5.1. Benchmark Families

We use two complementary Gaussian-mixture benchmark families:

**Model A** consists of random diagonal-anisotropic Gaussian-mixture targets with controlled radius, scale, and condition number. This family is used primarily for the deterministic VGS experiments. Its role is to provide a generic benchmark class in which protocol design can be studied without relying on highly structured low-dimensional symmetry.

**Model B** consists of structured grid/hypercube-type Gaussian-mixture targets built from the lattice ${\left\{-a,0,a\right\}}^{d}$. In d=2, we use the regular 3×3 model with nine equally weighted isotropic Gaussian modes placed on ${\left\{-a,0,a\right\}}^{2}$. In higher dimensions, we generalize this construction by selecting a prescribed number of modes uniformly and at random from the hypercube candidate set ${\left\{-a,0,a\right\}}^{d}$. This family is used for the sharpness-based experiments and for deterministic hypercube-type scaling studies. Its main advantage is interpretability: it supports both visually compelling low-dimensional illustrations and moderate-dimensional studies with controlled geometric structure.

#### 3.5.2. Optimization Workflow

In all experiments, we begin by scanning the one-parameter family of constant schedules β(t)≡β. This preliminary scan serves two purposes: it exposes the baseline objective landscape, and it identifies a stable warm start for subsequent refinement.

Starting from the best constant schedule, we refine the protocol hierarchically through

L=1→2→4→8,

where *L* denotes the number of temporal subintervals in the PWC representation. At each level, the solution from the previous coarser level is prolonged to the finer grid and then improved by a lightweight coordinate-descent outer loop. This continuation-style strategy is central to the practical robustness of the implementation: it avoids entering a high-dimensional protocol space too early, while still allowing nontrivial temporal structure to emerge.

The use of coordinate descent is pragmatic rather than fundamental. The present torch-based implementation makes gradient-based optimization a natural future direction, but the current lightweight gradient-free approach is sufficient for the present study and has the advantage of remaining transparent and stable across all reported experiments.

#### 3.5.3. Deterministic and Stochastic Evaluation

The evaluation methodology follows directly from the one-time vs. two-time distinction above.

For deterministic objectives, such as the VGS functional, the relevant quantities can be expressed through the time-marginal density and are therefore evaluated directly from exact marginals on moderate time grids. This is what makes the deterministic experiments particularly transparent: the objective is computed without Monte Carlo noise, so the resulting optimization can be interpreted directly in terms of target geometry and protocol structure.

For stochastic temporal-memory objectives, such as the sharpness criteria, the relevant observables depend on two-time correlation structure and do not reduce to one-time exact-marginal quantities. These are therefore estimated from controlled Monte Carlo trajectory simulations, typically using common random numbers to reduce variance across schedule comparisons.

Thus, the overall workflow is as follows:

- Specify a Gaussian-mixture target and a low-dimensional PWC protocol family;
- Scan the constant-β family to expose the baseline objective landscape;
- Use the best constant schedule as a warm start for hierarchical refinement 1→2→4→8;
- Evaluate deterministic one-time diagnostics from exact marginals and stochastic two-time diagnostics from controlled simulation when needed;
- Optimize the PWC coefficients with a lightweight coordinate-descent routine;
- Interpret the resulting schedules through both scalar diagnostics and representative low-dimensional visualizations.

For the deterministic **Model A + VGS** experiments, the primary reported quantity is the dimension-normalized velocity-gradient-sensitivity objective (11). When useful, we also report temporal-memory summaries such as $t_{\mathrm{cross}}\left(\hat{A}=0.8\right)$, not as optimized quantities in this model family but as auxiliary descriptors of how the resulting protocol affects temporal organization.

For the stochastic **Model B + sharpness** experiments, the primary reported quantities are the sharpness (13) and its regularized version (14). These scalar diagnostics are supported by direct plots of $\hat{A}\left(t\right)$ and β(t), which are especially informative in the low-dimensional structured examples.

In the earlier version of the manuscript [12] we discussed a broader quality-of-sampling suite, including path cross entropy, cost-to-go decompositions, drift–diffusion balance, Langevin mismatch, speciation timing, and related quantities. We refer readers interested in such exploratory diagnostics to that earlier version.

#### 3.5.4. Experimental Scope

The experiments reported in this paper compare only the protocol families that remain central to the revised narrative:

- Constant β schedules;
- Deterministic VGS-optimized PWC schedules;
- Sharpness- or regularized-sharpness-optimized PWC schedules.

Constant schedules are not included merely as trivial baselines. They are a core part of the methodology: they expose the basic objective landscape; reveal whether the relevant objective favors small-, intermediate-, or large-β regimes; and generate the warm starts for hierarchical refinement. The optimized PWC schedules then show how additional temporal structure can be exploited beyond the constant family.

All of the experiments are designed to remain computationally controlled and laptop-feasible, not because of any intrinsic hardware limitation but because this is part of the methodological philosophy of the paper. The central point is to preserve as much analytic and computational control as possible while still allowing meaningful target-dependent protocol design.

## 4. Results

### 4.1. Model A: Deterministic VGS on Random Anisotropic GMMs

Model A uses the random diagonal-anisotropic benchmark family with fixed parameters K=4, radius 3.0, scale 0.6, and condition number 20.0. The main paper notebook studies d=2 and d=8, and the associated scaling notebook extends the deterministic analysis to d=4 and d=16. Constant-β scans are carried out over [0.05, 20], and hierarchical refinement uses the levels L=1,2,4,8.

**Figure 1:**
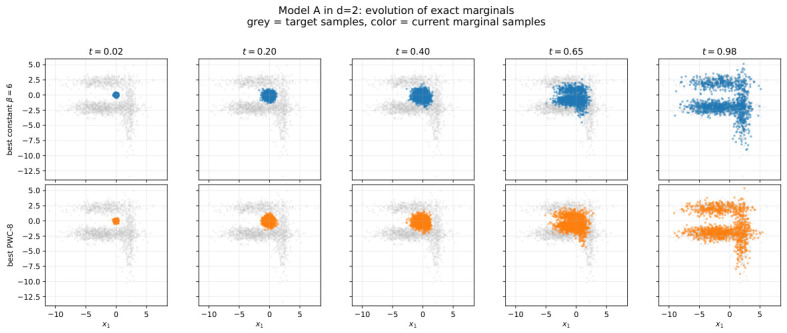
Model A in d=2: evolution of exact marginals under the best constant schedule (**top row**) and the best optimized PWC-8 schedule (**bottom row**). In each panel, grey points show samples from the target density, while colored points show samples from the current marginal at the indicated time. The top row corresponds to the best constant-β baseline, and the bottom row to the best hierarchical VGS-optimized PWC protocol.

Figure 1 gives a more dynamical view of the deterministic protocol-design problem in the representative d=2 Model A instance. The top row shows the evolution induced by the best constant schedule, while the bottom row shows the evolution under the best VGS-optimized PWC-8 protocol. In both cases, the target law is the same; what changes is the transient path through probability space. This is precisely the point of the protocol-design framework: even when the terminal target distribution is fixed, the intermediate marginal evolution can still be shaped in a nontrivial way. The comparison in Figure 1 makes this concrete. The optimized PWC protocol does not simply reproduce the best constant schedule with a slightly different scalar score; it induces a visibly different temporal organization of the marginal cloud as it evolves toward the target.

**Figure 2:**
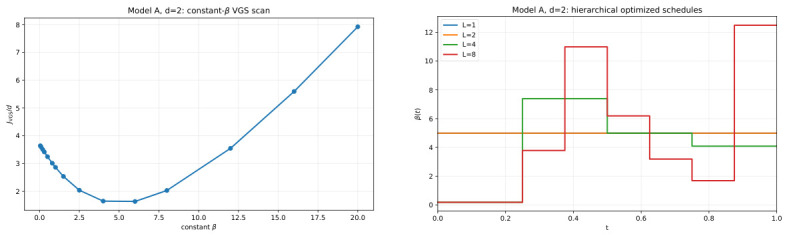
Model A in d=2: (**left**), constant-β scan of the deterministic VGS objective; (**right**), hierarchical optimized step-function schedules β(t) for L=1,2,4,8. The figure emphasizes that nontrivial optimized schedules emerge already in low dimensions.

Figure 2 summarizes the deterministic protocol-design logic for Model A in its simplest nontrivial setting. The left panel shows that even within the constant family β(t)≡β, the VGS objective has a clear and non-flat dependence on β, so the choice of protocol stiffness already matters at the one-parameter level. This constant-β scan therefore plays a dual role: it provides an interpretable baseline, and it identifies the warm start for subsequent refinement. The right panel then shows what is gained by allowing temporal structure. As the hierarchy progresses from L=1 to L=2,4,8, the optimized schedule departs from the constant baseline and develops nontrivial time dependence. Thus, the main lesson of Figure 2 is that deterministic protocol design is already meaningful in low dimensions: even when the terminal target law is fixed, the transient control field can be improved by introducing a modest amount of temporal flexibility into β(t).

The deterministic optimization logic remains meaningful beyond the low-dimensional visual example. For Model A, the d=8 study confirms that the same workflow—constant-β scan, warm start from the best constant schedule, and hierarchical refinement through L=1,2,4,8—continues to produce interpretable protocol structure in moderate dimensions. Therefore, the point of the d=8 experiment is not visual aesthetics but methodological robustness: the VGS-based protocol-design problem remains well posed, the exact-marginal deterministic evaluation remains available, and the optimized PWC schedule still improves on the best constant baseline.

Table 1 summarizes the main quantitative comparison for Model A. The d=2 and d=8 cases should be read together. The d=2 example shows most clearly that nontrivial optimized schedules emerge already in low dimensions, while the d=8 example shows that this is not merely a low-dimensional artifact. In both cases, the best PWC-8 schedule improves on the best constant schedule with respect to the deterministic VGS objective, while still remaining easy to interpret as a time-dependent refinement of the constant baseline. This is exactly the kind of behavior one would want from a protocol-design methodology: the optimization is strong enough to uncover nontrivial temporal structure, but controlled enough that the resulting schedules remain understandable rather than opaque.

A broader deterministic scaling study further supports this interpretation. In that study, the same Model A family was examined over d∈{2,4,8,16}, again using constant-β scans over [0.05, 20] and the same hierarchical 1→2→4→8 refinement strategy. The purpose of that study was not to claim a universal scaling law but to demonstrate that the deterministic evaluation pipeline remains computationally manageable, and that constant baselines remain meaningful across dimensions. We therefore view the scaling evidence as methodological rather than asymptotic: the exact-marginal VGS framework continues to function in a controlled way as *d* increases over the tested range. Additional scaling experiments involving changes in target complexity, including scans over the number of modes *K* for hypercube-type targets, are included in the accompanying notebooks, available at: https://github.com/mchertkov/ShortAdaPID (accessed on 19 April 2026).

Taken together, the Model A experiments support three conclusions: First, deterministic VGS optimization is already informative in low dimensions and produces nontrivial optimized PWC protocols. Second, the same protocol-design logic remains meaningful in moderate dimensions, as demonstrated explicitly in d=8. Third, the exact-marginal deterministic framework is computationally controlled enough to support systematic scans in dimension and in target complexity as well. This makes Model A the clean methodological backbone of this paper: it shows that protocol design can be posed, optimized, and interpreted at the density level without sacrificing computational tractability.

### 4.2. Model B: Sharpness-Based Optimization on Structured Grid/Hypercube GMMs

We now turn to Model B, where the emphasis shifts from deterministic control regularity to temporal organization, as measured by sharpness-based observables. In contrast to Model A, whose main role was methodological genericity, Model B is designed to expose how the geometry of the target distribution influences the optimization landscape itself. The experiments below therefore serve two related purposes: first, to show in a visually transparent setting that sharpness-based optimization can produce nontrivial time-dependent protocols; and second, to show that the resulting dependence on β is genuinely target-dependent and, hence, non-universal.

We begin with the regular 3×3 model in d=2, which is the clearest visual demonstration of the sharpness-based protocol-design idea. This is the natural place to explain the full logic of the optimization: we first scan the constant family β(t)≡β, then use the best constant schedule as a warm start, and finally refine hierarchically through L=1,2,4,8. Because the target geometry is so transparent in this case, both the scalar objectives and the evolving densities admit a direct visual interpretation.

**Figure 3:**
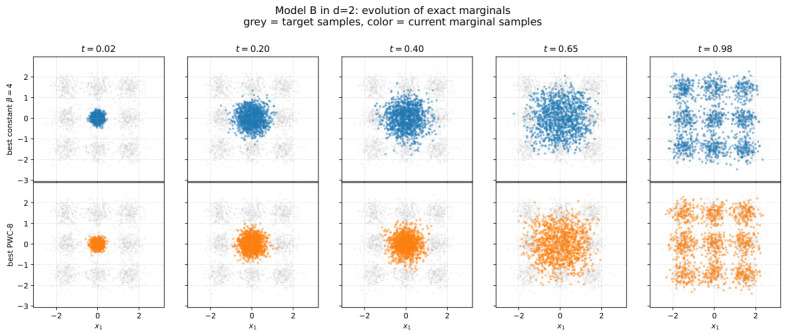
Model B in d=2: evolution of exact marginals under the best constant schedule (**top row**) and the best optimized PWC-8 schedule (**bottom row**). In each panel, grey points show samples from the target density, while colored points show samples from the current marginal at the indicated time. The top row corresponds to the best constant-β baseline, and the bottom row to the best regularized-sharpness-optimized piecewise-constant protocol.

Figure 3 provides the most direct dynamical view of what the sharpness objective is optimizing. The top row shows the evolution induced by the best constant schedule, while the bottom row shows the evolution under the best optimized PWC-8 protocol. In both rows, the terminal target law is the same; what differs is the transient organization of the marginal cloud. This is precisely the aspect of the dynamics that the sharpness objective is meant to probe. Thus, the optimized protocol is not merely better in the scalar objective but visibly different in how probability mass is reorganized in time on the way to the same final target. This figure is therefore best read as the qualitative, density-level counterpart of the scalar sharpness curves discussed next.

**Figure 4:**
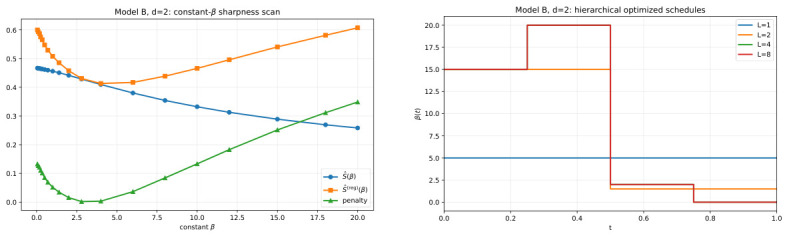
Model B in d=2: (**left**), $\hat{S}\left(\beta \right)$ and ${\hat{S}}^{\left(\mathrm{reg}\right)}\left(\beta \right)$ as functions of constant β; (**right**), hierarchical optimized step-function schedules β(t) for L=1,2,4,8. The figure emphasizes that regularized sharpness yields a nontrivial optimized PWC protocol in the visually interpretable d=2 setting.

Figure 4 explains how that nontrivial protocol emerges. The left panel shows that even within the one-parameter constant family, the sharpness landscape is already structured: neither $\hat{S}\left(\beta \right)$ nor ${\hat{S}}^{\left(\mathrm{reg}\right)}\left(\beta \right)$ is flat in β, and the regularized objective selects a meaningful operating region rather than a trivial boundary value. The right panel then shows that once temporal flexibility is introduced, the optimizer uses it: the protocol departs from the constant baseline and develops nontrivial time dependence under hierarchical refinement. The main lesson of Figure 4 is therefore the same as in the dynamical visualization, but now at the optimization level: the sharpness objective does not merely rank constant schedules; it supports genuinely time-structured protocol design.

**Figure 5:**
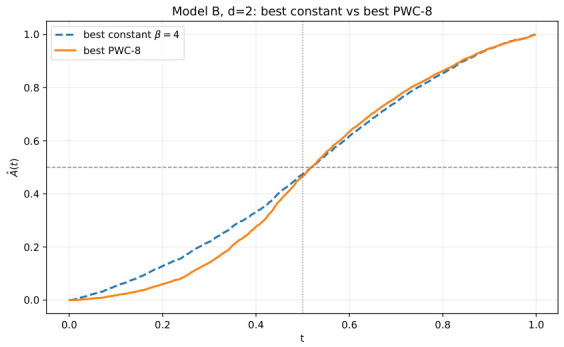
Model B in d=2: temporal-memory curves $\hat{A}\left(t\right)$ for the best constant schedule and the best optimized PWC-8 schedule under regularized sharpness.

Figure 5 makes the same point in the reduced one-dimensional temporal-memory observable. The optimized protocol does not simply lower ${\hat{S}}^{\left(\mathrm{reg}\right)}$ numerically; it changes the way temporal correlation builds up. This is important because it confirms that the sharpness-based optimization is not exploiting a numerical artifact of the scalar objective. Rather, it is selecting a protocol with a genuinely different temporal organization, and $\hat{A}\left(t\right)$ provides a compact summary of that difference. Taken together, Figure 3, Figure 4 and Figure 5 establish the main low-dimensional conclusion of this subsection: in the structured d=2 benchmark, regularized sharpness produces a nontrivial optimized protocol that is interpretable both geometrically and temporally.

We next turn to the hypercube-random K=9 case in d=8. This example plays a different role. It is not primarily a visual showcase but a test of target-dependence and non-universality. Here, the key object is the constant-β scan itself, because it reveals the shape of the sharpness landscape before any hierarchical refinement is attempted.

**Figure 6:**
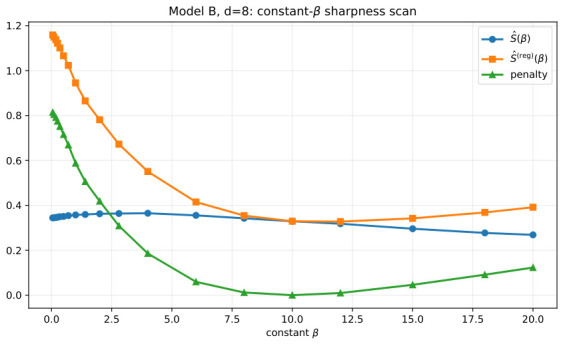
Model B in d=8: constant-β sharpness scans for the selected hypercube-random K=9 instance. This figure supports the claim that the β-dependence of sharpness is target-dependent and may involve competing small-β and large-β regimes.

Figure 6 shows that, in d=8, the dependence of both $\hat{S}\left(\beta \right)$ and ${\hat{S}}^{\left(\mathrm{reg}\right)}\left(\beta \right)$ on the constant schedule is nontrivial and can exhibit clear competition between small-β and large-β regimes. This is the essential evidence for our claim that the sharpness landscape is not universal. There is no single stiffness regime that dominates across targets. Instead, the shape of the objective depends substantially on the geometry of the target GMM. For the selected d=8 instance, the constant scan already reveals this competition, and this is the main message that we need from the higher-dimensional sharpness experiment in this paper.

As with Model A, we show only the most significant results. The full schedule plots, optimization traces, and auxiliary diagnostics are available at: https://github.com/mchertkov/ShortAdaPID (accessed on 19 April 2026).

The experiments discussed in this subsection therefore support two complementary conclusions: At the level of individual examples, the regular 3×3d=2 benchmark shows that regularized sharpness yields a genuinely nontrivial optimized PWC protocol, visible both in the evolving densities and in the temporal-memory curve $\hat{A}\left(t\right)$. At the collective level, the comparison between the d=2 and d=8 cases shows that sharpness-based protocol design is inherently target-dependent: the optimization landscape may favor different stiffness regimes for different targets, and the resulting protocol structure is not universal. We view this non-universality not as a weakness but as one of the main empirical insights of the Model B study. It suggests that temporal-memory-based objectives are sufficiently expressive to register meaningful geometric differences between targets, and that they therefore deserve broader systematic study in future work.

### 4.3. Deterministic Scaling on Hypercube-Type Targets

This subsection extends the reports of the two preceding subsections with the deterministic VGS study to hypercube-type targets and plays a supporting but important role in the experimental narrative. Its purpose is not to introduce a new objective but to test how the deterministic protocol-design pipeline behaves when the target geometry becomes more combinatorial and when either the ambient dimension or mode count is increased. In this sense, this subsection complements both of the earlier parts of the Results: it uses the deterministic VGS objective of Model A but applies it to the more structured hypercube-type targets associated with Model B.

Two families of scans are considered here: The first is a **dimension scan**, where we fix the number of modes at K=9 and vary the ambient dimension over d∈{2,4,8,16}. The second is a **mode-count scan**, where we fix the ambient dimension at d=8 and vary the number of modes over K∈{9,18,27,36,45}. In both families, the optimization workflow is the same as before: we first scan the constant family β(t)≐β, then use the best constant schedule as a warm start, and finally refine hierarchically through L=1,2,4,8 using coordinate descent. Thus, the only thing that changes across this subsection is the structure of the target family itself.

**Figure 7:**
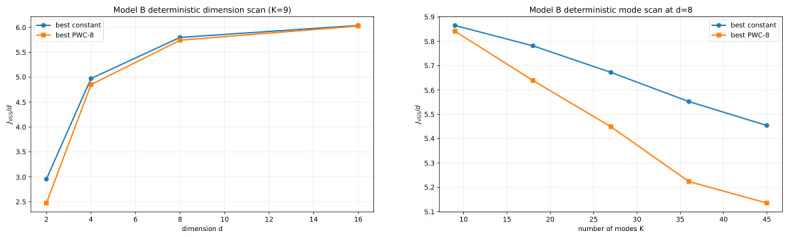
Deterministic VGS scaling on hypercube-type targets. (**Left**): best constant versus best PWC-8 for the dimension scan at fixed K=9. (**Right**): best constant versus best PWC-8 for the mode-count scan at fixed d=8. Together, the panels summarize the computationally controlled behavior of the deterministic protocol-design pipeline as either *d* or *K* increases.

Figure 7 summarizes the main deterministic scaling evidence. The left panel shows the effect of increasing ambient dimension at fixed target complexity K=9, while the right panel shows the effect of increasing target complexity at a fixed ambient dimension d=8. In both panels, the comparison is between the best constant schedule and the best optimized PWC-8 schedule under the deterministic VGS objective. The key qualitative message is that the optimization pipeline remains computationally controlled and interpretable in both directions of variation. As either *d* or *K* increases, the constant-schedule baseline remains meaningful, the hierarchical optimization remains executable, and the refined PWC protocol continues to provide a coherent comparison point.

At the same time, the purpose of Figure 7 is deliberately modest. We do not read these experiments as evidence for a universal asymptotic scaling law. Rather, we read them as evidence of methodological robustness. The exact-marginal deterministic framework can still be run on moderate-sized problems of varying structure, and the resulting optimization output remains interpretable enough to support systematic comparison. That is the main conclusion that this subsection is intended to support.

To keep the main paper focused, Figure 7 presents only the compact summary comparison. The full families of constant-β scans, hierarchical schedule profiles β(t), selected temporal-memory comparisons, and auxiliary tables for both scan families are not shown here but are available at: https://github.com/mchertkov/ShortAdaPID (accessed on 19 April 2026). Those Materials are useful for reproducibility and for readers interested in the detailed dependence of the optimized schedules on *d* and *K*, but they are not needed for the central narrative of this paper.

The collective conclusion of this subsection is therefore clear. The deterministic VGS framework is not confined to a single low-dimensional illustrative example. It remains computationally manageable under both increasing ambient dimension and increasing target complexity, at least over the experimentally tested range. This strengthens the broader conclusion already suggested by Model A: the proposed protocol-design methodology is not only interpretable in simple examples but also sufficiently robust to support structured scaling studies on more complex Gaussian-mixture targets.

## 5. Conclusions, Positioning, and Outlook

Harmonic PID provides a tractable and expressive framework for schedule design beyond terminal-law matching. The central contribution of this paper is to turn that tractable structure into a practical methodology for protocol optimization. Rather than treating the stiffness protocol β(t) as a fixed background ingredient, we treat it as a design variable that shapes the transient evolution of probability mass while preserving the terminal target law. This is the main conceptual shift of this paper.

Our methodological formulation relies on a clean distinction between two classes of observables. One-time quantities, such as velocity-gradient sensitivity, can be evaluated deterministically from exact time marginals in the Gaussian-mixture setting. In contrast, two-time quantities, such as temporal-memory observables derived from $\hat{A}\left(t\right)$, remain genuinely pathwise and must be estimated stochastically from trajectories. This separation is not merely technical; it is what allows us to combine exact-marginal deterministic optimization with controlled stochastic optimization in a single coherent schedule-design framework.

Our experiments support three main conclusions:

1. Nontrivial protocol design is already meaningful in the analytically controlled setting studied here. For Model A, deterministic VGS optimization produces non-constant optimized PWC schedules already in d=2, where the effect can be seen directly in the evolving exact marginals, and remains meaningful in d=8, where the main point is methodological robustness rather than visual intuition. This makes the exact-marginal VGS framework the clean backbone of this paper: it shows that protocol design can be posed, optimized, and interpreted at the density level without sacrificing computational tractability.
2. Sharpness-based objectives reveal a different and complementary aspect of the problem. In the structured d=2 Model B benchmark, regularized sharpness yields a genuinely nontrivial optimized protocol that is interpretable both geometrically and temporally. The evolving densities, the optimized β(t), and the corresponding $\hat{A}\left(t\right)$ curves all show that the protocol is not merely lowering a scalar objective numerically but is changing the temporal organization of the path itself. At the same time, the d=8 hypercube-random experiments show that the dependence on β is not universal: different target geometries may favor substantially different stiffness regimes, and in some cases there is visible competition between small-β and large-β behavior. We view this non-universality as one of the main empirical insights of this paper. It suggests that temporal-memory-based objectives are sensitive enough to register meaningful geometric differences between targets and, therefore, deserve broader systematic study.
3. The hierarchical constant-scan → warm-start →1→2→4→8 refinement strategy is effective and scalable enough for the present study. Across all reported notebooks, the same optimization logic is used successfully: first, scan the constant family, and then refine temporally through a small hierarchy of PWC schedules. This makes the optimization path interpretable and stable, and it provides a natural bridge between simple baselines and genuinely time-dependent protocols. The accompanying deterministic scaling study on hypercube-type targets further shows that this framework remains computationally manageable when either the ambient dimension or the target complexity is increased over the tested range.

In this sense, this paper is best positioned not as a claim about a single universally optimal schedule objective but as a methodology paper. Different objectives expose different aspects of the transient dynamics, and the appropriate notion of schedule quality is application-dependent. The exact-marginal VGS objective emphasizes control regularity and deterministic tractability. The sharpness-based objective emphasizes temporal organization and pathwise memory. The key point is that Harmonic PID is rich enough to support both viewpoints within a common analytically transparent framework.

Several directions for future work are immediate.

**Gradient-Based Outer Optimization:**

The present implementation uses hierarchical warm-started coordinate descent, which proved stable and sufficiently expressive for all experiments reported here. However, the codebase is already Torch-based, and a natural next step is to refactor the protocol-construction layer so that gradients with respect to the PWC coefficients propagate through the full evaluation pipeline.

**Richer Protocol Families:**

We restricted attention to low-dimensional PWC schedules because they provide a clean compromise between expressiveness and interpretability. A natural extension is to consider smoother or higher-resolution parameterizations of β(t), adaptive interval placement, or protocol families with explicit regularity constraints.

**Broader Objective Design:**

The present paper focuses on two complementary principles: deterministic velocity-gradient sensitivity, and stochastic temporal sharpness. A broader future program would develop additional objectives that connect more directly to specific application goals—for example, accelerated target assembly, robustness to multimodality, or controlled preservation/forgetting of temporal information.

**More Systematic Scaling and Geometry Studies:**

The current experiments already show that the target geometry matters, and that the resulting protocol landscape is not universal. A natural next step is therefore a more systematic empirical and theoretical study of how optimized schedules depend on the dimension, number of modes, anisotropy, and geometric arrangement of the target mixture components.

**Beyond Gaussian Mixtures:**

Gaussian-mixture targets are especially valuable because they preserve exact-marginal tractability, which is essential for the present methodological study. At the same time, one of the long-term goals is to transfer these schedule-design principles to broader classes of target distributions.

Overall, the main conclusion of this paper is that **the stiffness protocol** β(t) should be viewed as a meaningful design variable, not merely as a passive scalar regularization parameter. Even when the terminal target distribution is fixed, different protocol objectives lead to different optimized schedules and reveal different notions of what constitutes a “good” path to the same endpoint. Harmonic PID provides a rare setting in which such questions can be studied with both theoretical transparency and computational control, and this is what makes it a useful laboratory for schedule design in diffusion bridge-based generative modeling.

## Figures and Tables

**Table 1:** Model A comparison of the best constant schedule and the optimized PWC-8 schedule in d=2 and d=8. The optimized PWC protocol improves the deterministic VGS objective in both cases. Full optimized schedules are reported in the accompanying notebooks, available at: https://gihub.com/mchertkov/ShortAdaPID (accessed on 19 April 2026).

*d*	Best Const. β	Const. $J_{\mathrm{VGS}}/d$	PWC-8 $J_{\mathrm{VGS}}/d$	Improvement (%)	$t_{\mathrm{cross}}$ Shift
2	6.0	1.64027	1.51286	7.77	0.470→0.458
8	2.5	0.87640	0.84375	3.72	0.543→0.550

## Data Availability

In the interest of reproducibility, all code used to generate the figures and experiments reported in this paper is available at: https://github.com/mchertkov/ShortAdaPID (accessed on 19 April 2026).

## Appendix A. Path Integral Diffusion (PID)

This technical appendix recaps the linearly solvable Path Integral Diffusion (PID) framework [6]. Appendix A.1 clarifies how accounting for the time-dependent quadratic potential modifies the resulting dynamics.

**From Sampling to Stochastic Optimal Transport**

Let $x_{t}\in R^{d}$ evolve according to the controlled SDE (2), and denote by $p_{t}$ its marginal density. We prescribe $p_{0}\left(x\right)=\delta \left(x\right)$ and $p_{1}\left(x\right)\propto \exp\left[-E\left(x\right)\right]$, and we pose the question of inferring the score field $u_{0\to 1}\left(\cdot \right)$. PID casts the search for *u* as the **stochastic optimal transport** (SOT) problem Equation (1), subject to the dynamics (2) and the terminal constraint $p_{1}\propto e^{-E}$. Throughout this section, we focus on the low-integrability setting of [6], where the gauge and drift vector fields vanish (A=f=0) and the potential is isotropic and quadratic, $V_{t}\left(x\right)=\beta x^{2}/2$ with β>0.

**Linearly Solvable Structure**

Problem (1) becomes tractable because the optimal score field can be expressed in closed form once two linear PDEs are solved:

(A1) $$\begin{array}{cc} -\partial_{t}G_{t}^{\left(-\right)}+V_{t}\left(x\right)\;G_{t}^{\left(-\right)} & =\frac{1}{2}\Delta_{x}G_{t}^{\left(-\right)},\;G_{1}^{\left(-\right)}\left(x;y\right)=\delta \left(x-y\right), \end{array}$$

(A2) $$\begin{array}{cc} \partial_{t}G_{t}^{\left(+\right)}+V_{t}\left(x\right)\;G_{t}^{\left(+\right)} & =\frac{1}{2}\Delta_{x}G_{t}^{\left(+\right)},\;G_{0}^{\left(+\right)}\left(x;y\right)=\delta \left(x-y\right). \end{array}$$

With $G^{\left(\pm \right)}$ at hand, we arrive at Equation (6). Because the control is recovered from linear PDEs, the problem is said to be linearly solvable [8].

**Constant Stiffness: Analytic Benchmark**

For $V_{t}\left(x\right)=\frac{1}{2}\beta x^{2}$ with non-negative constant β, the Green functions are Gaussian, and Equations (A1) and (A2) yield an explicit analytic score [6] (see Appendix A.1 for details):

(A3) $$\begin{array}{cc} u_{t}^{*}\left(x;\beta \right) & =b_{t}^{\left(-\right)}\hat{y}\left(t;x;\beta \right)-a_{t}^{\left(-\right)}x=-a_{t}^{\left(-\right)}\left(x-\tilde{y}\left(t;x;\beta \right)\right), \end{array}$$

(A4) $$\begin{array}{cc} \hat{y}\left(t;x;\beta \right) & =E_{y∼p^{\left(\mathrm{probe}\right)}\left(\cdot ;x;\beta \right)}\left[y\right],\;p^{\left(\mathrm{probe}\right)}\left(y;x;\beta \right)\propto \exp\left(-E(y)-\Delta (t;x;y;\beta )\right), \end{array}$$

(A5) $$\begin{array}{cc} \Delta (t;x;y;\beta ) & =\frac{K_{t}}{2}{∥y-\frac{b_{t}^{\left(-\right)}}{K_{t}}x∥}^{2}-\frac{d}{2}\log\left(\frac{K_{t}}{2\pi }\right),\;\mathrm{where}\;K_{t}≐c_{t}^{\left(-\right)}-a_{1}^{\left(+\right)}, \end{array}$$

(A6) $$\begin{array}{cc} a_{t}^{\left(+\right)} & =\sqrt{\beta }\coth\left(t\sqrt{\beta }\right),\;a_{t}^{\left(-\right)}\;=\;c_{t}^{\left(-\right)}\;=\;\sqrt{\beta }\coth\;\left(\left(1-t\right)\sqrt{\beta }\right),\;b_{t}^{\left(-\right)}\;=\;\frac{\sqrt{\beta }}{\sinh(\left(1-t\right)\sqrt{\beta })}. \end{array}$$

Note that, at β→0, Equation (A6) becomes

(A7) $$a_{t}^{\left(+\right)}=\frac{1}{t},\;a_{t}^{\left(-\right)}\;=\;c_{t}^{\left(-\right)}\;=b_{t}^{\left(-\right)}\;=\;\frac{1}{1-t}.$$

**Why Time–Dependent Stiffness?**

A fixed β cannot simultaneously encourage exploration at early times and enforce contraction near t=1. Allowing $\beta_{t}$ to vary introduces exactly this flexibility. Appendix A.1 derives the Gaussian Green functions for a general schedule $\beta_{0\to 1}$.

### Appendix A.1. Time-Dependent Quadratic Potential

Here, we continue to discuss the case with A=f=0 and isotropic quadratic/harmonic but **time-dependent** potential (4), where $\beta_{t}\geq 0$. This case is more general than the “low”-level integrability setting discussed in [6] and also briefly reviewed above. This case is of interest to study because of the intuition gained on the benefit of different but time-independent β. The intuition—applied to the goal of building the target distribution discussed in [6]—suggests that smaller β at the beginning and larger β closer to the end may be beneficial for building the target distribution gradually. Indeed, as we start at $x_{0}=0$, small β at early stages will encourage faster spread—this is the stage of exploration. On the contrary, when you are closer to the end, you want to limit exploration, thus increasing β and allowing the model to fine-tune—in this regime, the terminal term bias, guaranteeing that we are getting to the target distribution, will be even stronger. Actually, if true, this logic suggests that the largest *t* does not matter much, but we need to increase β at some intermediate times—to stabilize memorization—locking to the final image at the point of phase transition, which should probably happen somewhere in the middle of the time interval.

In this regime of an isotropic but time-dependent quadratic potential, the differential relations from Appendix B of [6] still apply, except that the constant stiffness β is now replaced by the schedule $\beta_{t}$. We therefore obtain (in the following, and when not confusing we drop dependence on β to simplify notations)

(A8) $$\begin{array}{cc} G_{t}^{\left(-\right)}\left(x;y\right) & \propto \exp\left(-\frac{a_{t}^{\left(-\right)}}{2}x^{2}+b_{t}^{\left(-\right)}x^{⊤}y-\frac{c_{t}^{\left(-\right)}}{2}y^{2}\right),\;G_{t}^{\left(+\right)}\left(y;0\right)\propto \exp\left(-\frac{a_{t}^{\left(+\right)}}{2}y^{2}\right), \end{array}$$

(A9) $$\begin{array}{cc} \mp {\dot{a}}_{t}^{\left(\pm \right)}+\beta_{t} & ={\left(a_{t}^{\left(\pm \right)}\right)}^{2},\;{\dot{b}}_{t}^{\left(-\right)}=a_{t}^{\left(-\right)}\;b_{t}^{\left(-\right)},\;{\dot{c}}_{t}^{\left(-\right)}={\left(b_{t}^{\left(-\right)}\right)}^{2}, \end{array}$$

where ∝ means that we dropped the *t*-dependent multiplier.

Expressions for $u_{t}^{*}\left(x;\beta_{0\to 1}\right)$, $\hat{y}\left(t;x;\beta_{0\to 1}\right)$, and $\Delta (t;x;y;\beta_{0\to 1})$—originally introduced in Equations (A3)–(A5) for the case of the constant β—stay exactly the same in the general case of the time-dependent $\beta_{0\to 1}$, provided that we substitute original explicit expressions for $a_{t}^{\left(\pm \right)},b_{t}^{\left(-\right)}$, and $c_{t}^{\left(-\right)}$ set in Equation (A6) by a generally implicit representation via Equation (A9).

Recall that the Green functions must satisfy the boundary conditions—$G_{1}^{\left(-\right)}\left(x;y\right)=\delta \left(x-y\right)$ and $G_{0}^{\left(+\right)}\left(y;0\right)=\delta \left(y\right)$, resulting in the following asymptotic requirements:

(A10) $$\begin{array}{cc} t\to 1^{-}: & \;a_{t}^{\left(-\right)},b_{t}^{\left(-\right)},c_{t}^{\left(-\right)}\to \frac{1}{1-t}, \end{array}$$

(A11) $$\begin{array}{cc} t\to 0^{+}: & \;a_{t}^{\left(+\right)}\to \frac{1}{t}. \end{array}$$

Note that combining Equation (A3) with the t→0 asymptotics (A10) and accounting for the fact that $x_{0}=0$, we estimate that at sufficiently small *t*, $u_{t}^{*}\left(x;\beta \right)\approx b_{0}^{\left(-\right)}\bar{y}$, where $\bar{y}=Ep_{1}\left[y\right]$ is the first moment of the target distribution (which we may assume to be known).

#### Piece-Wise-Constant *βt*: Analytic Solution

This case is advantageous, as it allows for a fully analytic solution. Let us split the time domain into *K* equally spaced time intervals and then use the piecewise-constant (PWC) profile:

(A12) $$t\in \left[\left(k-1\right)/K,k/K\right],\;k=1,\cdots ,K:\;\beta_{t}=\beta_{k}.$$

Then, assuming continuity in time, and taking into account the asymptotic behavior of $a_{t}^{\left(-\right)},b_{t}^{\left(-\right)},c_{t}^{\left(-\right)}$ at t→1 documented in Equation (A10) and of $a_{t}^{\left(+\right)}$ at t→0 documented in Equation (A11), we derive

(A13) $$\begin{array}{cc} \; & t\in \left[1-1/K,1\right]:\;a_{t}^{\left(-\right)}=c_{t}^{\left(-\right)}=\sqrt{\beta_{K}}\;\coth\;\left(\left(1-t\right)\sqrt{\beta_{K}}\right),\;b_{t}^{\left(-\right)}=\frac{\sqrt{\beta_{K}}}{\sinh\;\left(\left(1-t\right)\sqrt{\beta_{K}}\right)}, \end{array}$$

(A14) $$\begin{array}{cc} \; & t\in \left[(k-1)/K,\;k/K\right],\;k=K-1,\ldots ,1:\\ \; & \;a_{t}^{\left(-\right)}=\sqrt{\beta_{k}}\;\frac{\;a_{k/K}^{\left(-\right)}+\sqrt{\beta_{k}}\;\tanh\;\left(\sqrt{\beta_{k}}\left(k/K-t\right)\right)\;}{\;\sqrt{\beta_{k}}+a_{k/K}^{\left(-\right)}\;\tanh\;\left(\sqrt{\beta_{k}}\left(k/K-t\right)\right)\;},\\ \; & \;b_{t}^{\left(-\right)}=b_{k/K}^{\left(-\right)}\;\sqrt{\frac{\beta_{k}-{\left(a_{t}^{\left(-\right)}\right)}^{2}}{\beta_{k}-{\left(a_{k/K}^{\left(-\right)}\right)}^{2}}},\\ \; & \;c_{t}^{\left(-\right)}=c_{k/K}^{\left(-\right)}+\frac{{\left(b_{k/K}^{\left(-\right)}\right)}^{2}}{\beta_{k}-{\left(a_{k/K}^{\left(-\right)}\right)}^{2}}\;\left(a_{k/K}^{\left(-\right)}-a_{t}^{\left(-\right)}\right), \end{array}$$

(A15) $$\begin{array}{cc} \; & t\in \left[0,1/K\right]:\;a_{t}^{\left(+\right)}=\sqrt{\beta_{1}}\;\coth\;\left(t\sqrt{\beta_{1}}\right), \end{array}$$

(A16) $$\begin{array}{cc} \; & t\in \left[k/K,(k+1)/K\right],\;k=1,\ldots ,K-1:\\ \; & \;a_{t}^{\left(+\right)}=\sqrt{\beta_{k+1}}\;\frac{\;1+\rho_{k+1}\left(t\right)\;}{\;1-\rho_{k+1}\left(t\right)\;},\;\rho_{k+1}\left(t\right)=\exp\;\left(-2\sqrt{\beta_{k+1}}\;\left(t-k/K\right)\right)\;\frac{a_{k/K}^{\left(+\right)}-\sqrt{\beta_{k+1}}}{a_{k/K}^{\left(+\right)}+\sqrt{\beta_{k+1}}}. \end{array}$$

**Remark on how the formulae are derived and stated.** For the “minus” branch $\dot{a}=a^{2}-\beta$, the fractional-linear variable $u=\left(a-\sqrt{\beta }\right)/\left(a+\sqrt{\beta }\right)$ satisfies $\dot{u}=+2\sqrt{\beta }\;u$, which yields the tanh/Möbius form used in (A14). For the “plus” branch $\dot{a}=\beta -a^{2}$, the same *u* obeys $\dot{u}=-2\sqrt{\beta }\;u$, giving the exponential Möbius map in (A16).

## Appendix B. Representations of Probability Densities

To set up a proper metric for this paper’s main goal—**choosing optimal** $\beta_{0\to 1}$—we utilize and juxtapose to each other the following (probability) densities, all defined for a given (fixed) $\beta_{0\to 1}$:

- The **target** density itself—$p^{\left(\mathrm{tar}\right)}\left(y\right)$, defined according to Equation (3) up to the normalization factor (partition function) by the explicitly given energy function E(·).
- Probability density of a **path** $p^{\left(\mathrm{path}\right)}\left(x_{0\to 1}|u_{0\to 1}^{*}\left(\cdot ;\beta_{0\to 1}\right)\right)$ generated according to the SODE (2), with $u_{0\to 1}\left(x_{0\to 1}\right)≐\left(u_{t}\left(x_{t}\right)|t\in \left[0,1\right]\right)$ substituted by the optimal protocol (conditioned to $\beta_{0\to 1}$) and defined in Equation (6). We will also be working with the **marginal** density of the state $x_{t}$ observed at the moment of time *t* and, thus, defined according to the following path integral:
  (A17) $$p^{\left(\mathrm{mar}\right)}\left(x_{t}|t;u_{0\to t}^{*}\left(\cdot ;\beta_{0\to 1}\right)\right)=\int p^{\left(\mathrm{path}\right)}\left(x_{0\to 1}|u_{0\to 1}^{*}\left(\cdot ;\beta_{0\to 1}\right)\right)Dx_{0^{+}\to t^{-}},$$
  where we utilize the standard (in theoretical physics) notations for the path integral differential:
  $$Dx_{0^{+}\to t^{-}}=\lim_{N\to \infty }\prod_{n=1}^{N-1}dx_{t_{n}},\;t_{n}=t\frac{n}{N}.$$
  Given dependence of the definitions of the path density and its marginals on the SODE (2), the densities will be represented empirically via generated sample paths.
- The density of the final state as **probe** at the time *t*, given that the system is observed in the state *x*, is another important object of the H-PID theory:
  (A18) $$p^{\left(\mathrm{probe}\right)}\left(\cdot |t;x;\beta_{0\to 1}\right)=\frac{q^{\left(\mathrm{probe}\right)}\left(\cdot |t;x;\beta_{0\to 1}\right)}{Z^{\left(\mathrm{probe}\right)}\left(t;x;\beta_{0\to 1}\right)}.$$
  Note that, according to Equations (8) and (9),
  $$\lim_{t\to 1}p^{\left(\mathrm{probe}\right)}\left(y|t;x;\beta_{0\to 1}\right)=\delta \left(x-y\right),\;\lim_{t\to 0}p^{\left(\mathrm{probe}\right)}\left(y|t;x;\beta_{0\to 1}\right)=p^{\left(\mathrm{tar}\right)}\left(y\right).$$
  In other words this density is certainly not a good approximation for the target density at larger *t*, and especially at t→1. This suggests that we should also consider **integrated probe** density
  (A19) $$p^{\left(i\text{-}\mathrm{probe}\right)}\left(\cdot |t;\beta_{0\to 1}\right)=\int p^{\left(\mathrm{mar}\right)}\left(x|t;u_{0\to t}^{*}\left(\cdot ;\beta_{0\to 1}\right)\right)\frac{q^{\left(\mathrm{probe}\right)}\left(\cdot |t;x;\beta_{0\to 1}\right)}{Z^{\left(\mathrm{probe}\right)}\left(t;x;\beta_{0\to 1}\right)}dx,$$
  for which the asymptotics become
  $$\lim_{t\to 1}p^{\left(i\text{-}\mathrm{probe}\right)}\left(y|t;\beta_{0\to 1}\right)=\lim_{t\to 0}p^{\left(i\text{-}\mathrm{probe}\right)}\left(y|t;\beta_{0\to 1}\right)=p^{\left(\mathrm{tar}\right)}\left(y\right).$$
- Let us define the **expectation** (average value) of the final state (conditioned to the observation of *x* at time *t*):
  (A20) $$\hat{y}\left(t;x;\beta_{0\to 1}\right)≐E_{y∼p^{\left(\mathrm{probe}\right)}\left(\cdot |t;x;\beta_{0\to 1}\right)}\left[y\right]=\int_{R^{d}}yp^{\left(\mathrm{probe}\right)}\left(y|t;x;\beta_{0\to 1}\right)dy.$$
  The expected final state was called an “order-parameter” in [6] because, in analysis of auto-correlations, it shows a sharper (than for the original *x*) transition from the de-correlated case to what seems to be relatively close structurally to the finite generated sample.
  Next, it is natural to introduce what we call **predicted** density of the **expected** state:
  (A21) $$\begin{array}{cc} p^{\left(\mathrm{pred}\text{-}e\right)}\left(y|t;\beta_{0\to 1}\right) & =\int \delta \left(y-\hat{y}\left(t;x;\beta_{0\to 1}\right)\right)p^{\left(\mathrm{mar}\right)}\left(x|t;u_{0\to t}^{*}\left(\cdot ;\beta_{0\to 1}\right)\right)dx \end{array}$$
  We will see below that these densities will be useful for understanding the quality of the final state as we **grow** it in time with H-PID. Let us now discuss how we can sample from $p^{\left(\mathrm{pred}\text{-}e\right)}\left(y|t;\beta_{0\to 1}\right)$ (or from $p^{\left(\mathrm{pred}\text{-}r\text{-}e\right)}\left(y|t;\beta_{0\to 1}\right)$). The sampling is done in two steps: first, sample a path and record the state *x* where it arrives at the moment of time *t*; and second, evaluate the function $\hat{y}\left(t;x;\beta_{0\to 1}\right)$ (or $\hat{y}\left(t;x;\beta_{0\to 1}\right)$) at these *t* and *x*.
- Gaussian density built from $\Delta (t;x;y;\beta_{0\to 1})$—which is a quadratic form in *x* and *y* with coefficients dependent on time, defined in Equation (9)—becomes
  (A22) $$\begin{array}{cc} \rho (y\;|\;t;x;\beta_{0\to 1}) & =\exp\;\left(-\Delta (t;x;y;\beta_{0\to 1})\right)=N\;\left(y\;|\nu_{t}\left(x\right)≐\frac{b_{t}^{\left(-\right)}}{K_{t}}x,\frac{1}{K_{t}}I_{d\times d}\right)\\ & =\exp\left(-\frac{K_{t}}{2}{∥y-\frac{b_{t}^{\left(-\right)}}{K_{t}}x∥}^{2}+\frac{d}{2}\log\left(\frac{K_{t}}{2\pi }\right)\right),\;K_{t}≐c_{t}^{\left(-\right)}-a_{1}^{\left(+\right)}. \end{array}$$
  Here, $a^{\left(+\right)}$, $a^{\left(-\right)}$, $b^{\left(-\right)}$, and $c^{\left(-\right)}$ are the time (and $\beta_{0\to 1}$)-dependent coefficients obtained by solving the coupled Riccati-type ODEs for the harmonic path integral (cf. Appendix A). As discussed in Appendix A.1, in the case of piecewise-constant $\beta_{0\to 1}$ these coefficients are computed exactly in each interval and matched continuously at the interval boundaries.
  We call the auxiliary Gaussian density ρ(·∣·;·;·) the **re-weighting** density because of its role played in Equation (8)—re-weighting from the target to probe densities.

## Appendix C. Gaussian Mixtures

We consider the special case when the target distribution is a Gaussian mixture with component–specific full covariances:

(A23) $$p^{\left(\mathrm{tar}\right)}\left(y\right)\;\to \;p^{\left(\mathrm{tar}\text{-}G\text{-}\mathrm{Mix}\right)}\left(y\right)\;≐\;\sum_{n=1}^{N}\varrho_{n}\;N\;\left(y;\mu_{n},\Sigma_{n}\right),\;\sum_{n=1}^{N}\varrho_{n}=1,$$

where each $\Sigma_{n}\in R^{d\times d}$ is symmetric positive definite.

The **re-weighting density** ρ(y∣t;x), defined in Equation (A22) (restated here for convenience),

$$\rho \left(y\;|\;t;x\right)=N\;\left(y\;|\;\frac{b_{t}^{\left(-\right)}}{K_{t}}x,\;\frac{1}{K_{t}}I_{d}\right),$$

is isotropic with scalar gain $b_{t}^{\left(-\right)}$ and precision $K_{t}=c_{t}^{\left(-\right)}-a_{1}^{\left(+\right)}>0$ determined by the chosen $\beta_{0\to 1}$–protocol, with $a_{t}^{\left(\pm \right)},b_{t}^{\left(-\right)},c_{t}^{\left(-\right)}$ solving ODEs (A9) subject to the boundary conditions Equations (A10) and (A11).

**Probe Density as a Product of Experts**

The **probe** (a product of experts: target × re-weighting) is defined by Equations (8) and (A18) and restated as

$$p^{\left(\mathrm{probe}\right)}\left(y|t;x\right)=\frac{q^{\left(\mathrm{probe}\right)}\left(y|t;x\right)}{Z^{\left(\mathrm{probe}\right)}\left(t;x\right)}=\frac{p^{\left(\mathrm{tar}\right)}\left(y\right)\;\rho \left(y\;|\;t;x\right)}{\int p^{\left(\mathrm{tar}\right)}\left(y^{\prime }\right)\;\rho \left(y^{\prime }\;|\;t;x\right)\;dy^{\prime }}.$$

Plugging in (A23) and ρ yields the (unnormalized) mixture–Gaussian product

(A24) $$\begin{array}{cc} q^{\left(\mathrm{probe}\text{-}G\text{-}\mathrm{Mix}\right)}\left(y|t;x\right)\; & \propto \;N\;\left(y\;|\;\nu \left(t;x\right)=\frac{b_{t}^{\left(-\right)}}{K_{t}}x,\;\frac{1}{K_{t}}I_{d}\right)\;\sum_{n=1}^{N}\varrho_{n}\;N\;\left(y;\mu_{n},\Sigma_{n}\right),\\ & \propto \sum_{n=1}^{N}\varrho_{n}\;w_{n}\left(t;x\right)\;N\;\left(y;{\tilde{\mu }}_{n}\left(t;x\right),{\tilde{\Sigma }}_{n}\left(t\right)\right), \end{array}$$

where the newly introduced variance, mean, and mixing weights are

(A25) $$\begin{array}{cc} {\tilde{\Sigma }}_{n}\left(t\right) & \;≐\;{\left(\Sigma_{n}^{-1}+K_{t}\;I_{d}\right)}^{-1},\;{\tilde{\mu }}_{n}\left(t;x\right)\;≐\;{\tilde{\Sigma }}_{n}\left(t\right)\left(\Sigma_{n}^{-1}\mu_{n}+b_{t}^{\left(-\right)}\;x\right), \end{array}$$

(A26) $$\begin{array}{cc} w_{n}\left(t;x\right)\; & ≐\;N\;\left(\nu \left(t;x\right);\;\mu_{n},\;\Sigma_{n}+\frac{1}{K_{t}}I_{d}\right)\\ & =\frac{\exp\;\left(-\frac{1}{2}{\left(\nu \left(t;x\right)-\mu_{n}\right)}^{⊤}{\left(\Sigma_{n}+\frac{1}{K_{t}}I_{d}\right)}^{-1}\left(\nu \left(t;x\right)-\mu_{n}\right)\right)}{\sqrt{{\left(2\pi \right)}^{d}\;\det\;\left(\Sigma_{n}+\frac{1}{K_{t}}I_{d}\right)}}. \end{array}$$

(Equation (A24) is a an example of the Gaussian product identity in action.)

Then **predicted (expected) state map**, defined in (A20), also becomes a Gaussian mixture

(A27) $$\begin{array}{c} \hat{y}\left(t;x\right)\;=\;\frac{\int y\;q^{\left(\mathrm{probe}\text{-}G\text{-}\mathrm{Mix}\right)}\left(y|t;x\right)\;dy}{\int \;q^{\left(\mathrm{probe}\text{-}G\text{-}\mathrm{Mix}\right)}\left(y|t;x\right)\;dy}\;=\;\frac{\sum_{n=1}^{N}\varrho_{n}\;w_{n}\left(t;x\right)\;{\tilde{\mu }}_{n}\left(t;x\right)}{\sum_{n=1}^{N}\varrho_{n}\;w_{n}\left(t;x\right)}. \end{array}$$
